# Role of Dietary Fiber in Poultry Nutrition

**DOI:** 10.3390/ani11020461

**Published:** 2021-02-09

**Authors:** Oscar J. Tejeda, Woo K. Kim

**Affiliations:** Department of Poultry Science, University of Georgia, Athens, GA 30602, USA; oscar.tejedamartinez@uga.edu

**Keywords:** dietary fiber, broiler chicken, intestinal morphology, nutrient digestibility, digestive organ

## Abstract

**Simple Summary:**

Dietary fiber is an inherent compound found in common vegetables that are fed to broiler chickens. Fiber has the ability to scape digestion and absorption in the small intestine, which makes it able to affect the way other nutrients are absorbed and metabolized in the gastrointestinal tract. The functionality attributed to fiber varies based on chemical and physical structure, and most of the time, it is hard to make a clear differentiation among attributes due to the complexity of carbohydrates found in common feedstuffs. Data on the effect of dietary fiber have been gaining importance due to the use of grains for ethanol production and the search for feed alternatives that could help in sustainable and cost-effective broiler production. Therefore, it is paramount to integrate the current knowledge on the nutritional and physiological attributes of dietary fiber in poultry diets to be able to make correct use of fibrous feedstuffs.

**Abstract:**

Dietary fiber (DF) is an intrinsic component in plant feedstuffs that has been associated with physiological, structural, and functional changes in the gastrointestinal tract. DF is composed of non-starch polysaccharides (NSP), oligosaccharides, and lignin that scape digestion and enzymatic hydrolysis. In general terms, fiber can be classified as insoluble or soluble based on their solubility in water. Both fiber types have direct nutritional implications in broiler diets. Inclusion of insoluble DF in broiler diets modulates intestinal morphology, digestive organ development, nutrient absorption, growth performance, and intestinal microbiota. Soluble DF is thought to increase intestinal viscosity and is associated with negative changes in intestinal microflora and reduction in nutrient absorption. Nevertheless, there is a group of soluble fibers, integrated by oligosaccharides, that function as prebiotics positively modulating intestinal microbiota. Due to the changes in chemical structure and subsequent variation in functionality, it is a difficult task to assign clear attributes to DF as a whole. Therefore, the following review paper compiles data from research conducted using DF and tries to unify such information into practical decisions to be considered when using DF as a functional nutrient in poultry nutrition.

## 1. Introduction

Carbohydrates represent the biggest constituent of poultry diets and are one of the least understood feed components in broiler diets; this is especially so for dietary fiber (DF). Different reports regard DF either as a functional component for normal digestive organ functioning [1,2,3] or as an antinutrient [4,5,6]. This is likely due to the complexity and variability of DF’s physical and chemical structure, which makes this portion of the diet more challenging to understand chemically and physiologically [7]. From a nutritional perspective, DF has been defined as the group of heterogenous compounds, including all the saccharides (excluding starch, i.e., oligosaccharides, polysaccharides, pectins, gums, waxes) and lignin that are resistant to enzymatic hydrolysis [8,9]. A simpler definition of fiber describes DF as the sum of soluble and insoluble non-starch polysaccharides (NSP) and lignin [7]. Notwithstanding their composition, soluble fibers are avoided when formulating broiler diets since these are the type of fibers that increase intestinal viscosity, reducing the passage rate of the digesta through the gastrointestinal tract, which can create hypoxic conditions in the intestinal tract that favor pathogenic bacteria growth [10,11]. On the other hand, insoluble fibers have been used as feed diluents in monogastric diets because their physical and chemical structures allow them to be inert when mixing with the intestinal bolus [1,5]. There has been a substantial variability in the results obtained when assessing the impact of dietary fiber in poultry nutrition due to differences in fiber type and diet formulation [12]. Both soluble and insoluble fibers have practical implications for the feed and monogastric animal industries, especially with the current increase in the utilization of alternative feedstuffs high in fibers, which makes the understanding of the functional role of different DF in poultry nutrition a paramount step for production efficiency. Therefore, the present review focuses on the potential mode of action of dietary fiber in nutrient digestibility, modulation of organ growth, intestinal morphology modulation, regulation of intestinal microflora, and health in poultry, and it asks how to face both opportunities and obstacles when using dietary fiber.

## 2. Effect of Dietary Fiber in Poultry Diets

The unique ability of fiber to escape digestion and absorption provides the opportunity to regulate intestinal morphology [13,14], interact with nutrients of the digesta [15], interact with intestinal microflora [16,17], and modulate general digestive organ activity [18,19], resulting in changes in nutrient utilization and growth performance. It has also been reported that different components of DF can modulate the physiological structure and functionality of the gastrointestinal tract differently [6,18,20]. All these changes present an overall modulation of the nutrient metabolism that might result in impacts on performance. The way dietary fibers exert their effects seems to be related with changes in morphology, organ growth, general nutrient digestibility, and microbiota. Such parameters will be discussed herein.

### 2.1. Intestinal Morphology

Poultry require a certain amount of DF for normal intestinal physiology functions to take place [21]. The mechanisms by which DF functions in the gastrointestinal tract depend on the chemical structure, particle size, and amount being used [12,22,23]. Across poultry species, a rapid and relatively consistent intestinal response to changes in DF—resulting in modification of intestinal length, villus height, crypt depth as well as the passage rate and size through different segments of the intestines—has been reported [1,13,24,25]. The improvements in villus height and overall epithelial cell arrangement have been regarded as desirable due to the potential increase in nutrient absorption. Such changes are, more often than not, seen when feeding fibers in most poultry species (Table 1). In a study [13], it was shown that feeding isonitrogenous and isocaloric diets with increments of crude fiber from 2.8 to 9% to turkeys resulted in an increase in the number and size of villi in all sections of the small intestine with higher fiber-containing diets. Similar results have been reported in quails [14] fed 1.5% micronized wheat fiber, which usually results in an increase in relative length of intestinal segments, villi height, villus thickness, and villi to crypt proportions. In geese, increases in villi height were reported [25] with inclusions of alfalfa, rice hulls, or pectins; no changes with inclusion of barley hulls or cellulose; and reductions in villi height with inclusions of lignin. However, the inherent increment in nutrients for the maintenance of such tissues is generally ignored [26]. In a study [27], it was reported that inclusion of 8% cellulose to broiler diets resulted in higher crude protein and amino acid (i.e., Glu, Asp, and Thr) losses compared to diets fed 3% cellulose. It is important to bear in mind that such endogenous losses might not be from specific endogenous loses but also from dietary loses. However, there is a lack of research in this area.

It has been pointed out that the increase in inevitable crude protein and amino acid endogenous loses in broilers fed high fiber levels [27]. Therefore, an important question to ask is whether improvements in villi height should be considered as an advantage because more villi height, in theory, more absorptive capacity; or should such improvements be considered a burden because more villi height leads to more cell turnover, which might increase the requirements for amino acids important for intestinal functionality (i.e., threonine, aspartic acid, glutamic acid, proline). In such a case, adequate nutrient matrix modifications should be made to offset for such changes and ensure maximum growth.

### 2.2. Organ Growth

Poultry species have a characteristic digestive tract composed of beak, esophagus, crop, proventriculus, ventriculus (gizzard), and small and large intestine. Proventriculus, gizzard, and the intestines play the role of digestion and absorption and are, thus, the most influenced by dietary changes [32]. The proventriculus is were hydrochloric acidis secreted, but due to its small volume, the majority of mechanical digestion takes place in the gizzard [33]. Nevertheless, fiber inclusion affects the proventriculus and gizzard in conjunction (Table 1). One important role of the gizzard is to regulate digesta particle size in the gastrointestinal tract [2,33] with the ability to sense and modulate the passage of feed from the upper digestive tract to the small intestine based on particle size. Factors such as fiber type and particle size are determinant factors that stimulate the muscular activity of the gizzard, resulting in increased size [4]. The normal retention of feed in the gizzard has been shown to be between half an hour to one hour, which can increase up to two hours when structural (i.e., fiber) components are added to the diets [34]. In an experiment [28], it was reported that inclusion of 6% wood shavings increased the size of the proventriculus and gizzard while reducing the relative empty weight of the small intestine and increasing feed efficiency by 4.7%. Similarly, studies using oat hulls and soyhulls at 3% in the diet have been shown to result in increased proventriculus and gizzard size as well as in improved feed conversion [21]. The increase in particle size and fiber in the diet increases the muscular activity of the gizzard as a consequence of the need for particle size reduction, resulting in heavier weights as observed by different researchers in different poultry species [14,21,28]. The increase in the size of the proventriculus and gizzard is a logical result of an increased volume due to the slower passage rate of the almost-intact feed particles, which can only be solved by muscular grinding in the gizzard.

The presence of insoluble dietary fiber such as cellulose, lignin, and arabinoxylans can also modulate the size of the small intestine, pancreas, and ceca, which can result in improvements of the total tract apparent retention of nutrients and feed efficiency as described by different researchers [15,21,35,36]. In an experiment [13], the authors observed that turkey hens fed 6% and 9% crude fiber had a reduction of digestibility of crude protein, fat, and gross energy during the first 4 weeks of age which disappeared at the end of the eighth week. In fact, at the end of the experiment (week 14) such birds had an improved body weight compared to the control group (group fed 3% CF). Such changes were due to the prompt ability of the gastrointestinal tract to compensate for changes in dietary fiber, thus increasing the ability to use nutrients. One of the targets when using insoluble dietary fiber is to increase pancreatic secretions (i.e., amylases, lipases, proteases) that can improve substrate breakdown and subsequent release of nutrients. It has been reported that additions of insoluble fibers at 1% in diets of pullets can increase the relative weights of proventriculus, gizzard and liver and improve pancreatic proteolytic activity [37]. Similarly, chickens fed 3% wheat bran have shown increased relative weights of gizzard, small intestine, and pancreatic amylase and trypsin activity that was correlated with increased nutrient digestibility [38]. The presence of such indigestible carbohydrates (i.e., cellulose, arabinoxylans) and other indigestible plant components (i.e., lignin) upregulate digestion activity as a means to compensate for the reduced hydrolysis of glycosidic bonds among molecules, resulting in an increased nutrient breakdown (digestibility) of others (i.e., starches, protein) [2]. Therefore, insoluble fiber with particle size bigger than 1.5 mm can help in the stimulation of digestive organ growth with potential changes in nutrient digestibility.

### 2.3. Nutrient Digestibility

In most poultry research, insoluble dietary fiber has been used as nutrient diluent due to the lack of enzymes to digest β 1–4, β 1–3, and β 1–6 linkages found in such non-starch polysaccharides [39] which have been regarded as impairing performance when used in high amounts due to a slowing down and dilution of nutrient intake [40]. As a consequence, commercial diets are generally formulated to contain a maximum of 2–3% CF [7]. However, inclusion of specific insoluble fiber types such as cellulose at 3–5% in the diet has often proven to improve nutrient utilization. DF can also increase pancreas enzymatic activity and reverse peristalsis that can lead to an increase in nutrient digestibility [5,15,28]. The reverse peristalsis causes bile salts to reach the gizzard, where the bolus is being mixed with gastric secretions. This results in an improved fat emulsification, reducing the potential of fat droplets to coat nutrients, and as a consequence, nutrients are more readily hydrolyzed and absorbed [2]. However, the results obtained when using dietary fiber can be heavily impacted by the source of fiber and the formulation of iso-nitrogenous and iso-caloric diets (Table 2).

Inclusion of insoluble fibers such as cellulose and lignin from plant sources at 3–5% in the diet is commonly known to improve nutrient metabolism due to their ability to modulate gastric secretions from the proventriculus and muscular activity from the gizzard [30,33]. The gizzard is a grinding organ equipped with both large and small muscles. The grinding is performed by larger muscles, whereas smaller muscles are in charge of positioning the luminal contents for particle size reduction and gastric digestion. The movement of the digesta out of the gizzard is based on particle size, which is controlled by the small openings of the pylorus, which functions as a sieve [33]. Regardless of the initial size, the organic feed components leaving the gizzard have a consistent particle size range [2]. It would follow that larger particles of DF will help in the retention of bolus in the upper portion of the gastrointestinal tract, slowing down the passage rate and increasing the exposure of feed components to HCl and enzymes from the proventriculus. This results in the accumulation of insoluble fiber in the gizzard and increases the gastroduodenal reflux and subsequent digestibility of nutrients [2,30]. Insoluble dietary fiber has been shown to modulate (oftentimes positively) digestion of starches [28], fats [3], and crude proteins [1] when added at 3–5% in the diet.

Soluble fibrous components of the diet such as pectins and arabinoxylans have been regarded to increase intestinal viscosity, reducing the absorption of nutrients [43] and modulating digesta passage rate, which creates environments full of substrates for microbial growth [5,44]. Viscosity-forming soluble fibers such as β-glucans, pectins, and arabinoxylans have the ability to interact with water molecules [45], slowing down the passage rate in the small intestines, reducing enzyme diffusion and subsequent substrate breakdown, and increasing the free nutrients in the intestinal lumen, which favors the establishment of pathogenic bacteria that have been regarded as playing a critical role in the competition for nutrient utilization with the host [43]. In a study [42], apparent digestibility of lipids, protein, and metabolizable energy showed a linear decrease when feeding 0, 1, and 3 g/kg of guar gum to broiler chickens, decreasing feed efficiency by 4% when fed at 3 g/kg. Inclusion of soluble fiber such as high-methylated pectins reduced feed efficiency up to 28% when provided in diets at 3% [31]. Therefore, soluble viscous-forming fibers are undesirable at any levels in diets of broilers due to negative impacts in nutrient digestibility.

Two of the most prominent factors affecting digestion efficiency of nutrients in the presence of soluble fiber are solubility and fermentability because of their impact on passage rate in the small intestines and the fermentability in the hindgut, respectively [46,47]. Both of these factors are determined by the type of linkages and the amount of branching among sugar units, which allows or prevents interactions with water molecules and/or potential bacterial break down [45]. It is accepted that long β 1–4 chains, such in the case of cellulose, are poorly soluble, whereas β 1–3 branches are highly soluble, such in the case of β-glucans [48]. In poultry nutrition, the term “water-soluble carbohydrate” has been erroneously interchanged with the term “antinutritional fiber”. Even though most of the soluble fibers have the ability to form viscosity in the presence of water, there is a small group of soluble fibers that do not. In fact, low-molecular weight carbohydrates such as oligosaccharides are regarded as prebiotics that facilitate the growth of beneficial bacteria from which Lactobacillus spp. and Bifidobacterium spp. have been targeted as beneficial for intestinal development [49,50]. Therefore, the hygroscopic properties of some oligo- and polysaccharides should not necessarily be directly associated with anti-nutritional factors.

The difference in how soluble and insoluble fiber affect intestinal passage rate relies on the site of action of each fiber type. When insoluble fiber is fed as particles bigger than 1.5 mm, it can accumulate in the upper part of the gastrointestinal tract (i.e., gizzard and duodenum loop), where most of the bolus mixes with enzymes and where mechanical grinding takes place (in the gizzard) [51]. While small (3–5%) additions of insoluble fibers can improve nutrient digestibility, extreme supplementation can interrupt normal digestion metabolism by the formation of coating structures that reduce the accessibility of digestive enzymes to nutrients [52,53]; therefore, it is unclear how the threshold for excess DF should be defined. Type and source of fiber, as well as other parameters intrinsic to diet formulation, may influence this threshold. Finally, it is paramount to bear in mind that fiber should be used as a functional nutrient and not as a nutrient per se, and the adequate nutritional amendments should be made when using fibrous feedstuffs in terms of energy, protein, and their ratios.

### 2.4. Dietary Fiber and Intestinal Microflora Activity

After the bacterial inoculum introduced at hatch, the diet plays the most crucial role in determining the composition and density of the intestinal microflora [54]. As specific bacterial species have substrate preferences, it would follow that bacterial populations in the intestines are influenced by changing the diet [55]. The ceca is considered the main site of bacterial activity in the gastrointestinal tract in poultry and is, generally, the organ used for determination of bacterial populations in broilers [56]. The carbohydrate fraction is the most important dietary component regulating the intestinal microbial activity in broilers, particularly with regards to DF, which escapes digestion [57,58]. The magnitude of the effects of the dietary carbohydrates depends on the type and amount of carbohydrate. Most data have indicated that water-soluble NSP are the most influential compounds, as these can be degraded to be utilized as substrate by intestinal bacteria (Table 3) [44,58]. These soluble components provide the energy for bacteria, allowing them to use other nutrients (i.e., nitrogen) as substrates for the production of metabolites. It is clear that the presence of viscous-forming carbohydrates in the digestive tract have adverse effects on performance [31], but the presence of bacteria appears to aggravate the problem. In a study [10], it was observed that germ-free broilers fed methylated citrus pectin were not strongly affected in terms of ileal digestibility of starch and energy compared to conventional broilers. Therefore, it is thought that the negative effects of water-soluble carbohydrate on performance and general metabolism in broiler is worsened by intestinal microflora and not only by intestinal viscosity.

Feed ingredients affect bacterial populations differently depending on the type and length of carbohydrates that they are made of. In a study [57], it was observed that barley and rye tend to favor the development of pathogenic bacteria (i.e., Clostridium coccoides) and reduction of beneficial bacteria (i.e., Bifidobacterium sp.) when compared to groups containing enzyme addition. This difference can be explained by the fact that soluble fiber is generally associated with imbalances in microflora, favoring anaerobic pathogens that compete with the host for the uptake of nutrients [59,60]. Even though all viscous NSP are deemed as soluble, not all soluble NSP should be deemed as viscous (antinutritional). Groups of low-molecular weight compounds such as oligosaccharides and fructans are highly soluble and fermented by microbiota in the large intestine of broilers, and these can be used to generate volatile fatty acids and other beneficial chemical compounds [7,61]. In fact, such low-molecular weight carbohydrates are frequently used as prebiotics to promote the growth of beneficial bacteria in the intestines [62]. Soluble NSP such as β-glucans have been shown to positively alter the expression of immune genes associated with T helper type-1 cells, resulting in downregulation of nitric oxide synthase, interleukins, and gross lesion severity in birds infected with Eimeria [63].

A balanced microflora in healthy broilers has the ability to produce a diverse number of metabolic end products including antigenotoxic compounds and short-chain fatty acids (SCFA) [64]. There are different SCFA that can be synthesized in the ceca, including acetic, propionic, and butyric acid [65]. The type and quantity of fiber and other undigested dietary compounds reaching the posterior gut are the main factor determining the type of bacteria and the type of metabolite being produced. Among these metabolites, butyric acid has been regarded as the most beneficial SCFA due to its antimicrobial and anti-inflammatory properties [66], as well as its use as an energy source by epithelial cells [67]. Fermentation of fiber in the gastrointestinal tract has been associated with increases in butyric acid, which may serve as a source of energy for enterocytes or as an antimicrobial for pathogenic bacteria [68]. Therefore, the production of butyric acid may lead to promotion of intestinal health.

### 2.5. Growth Performance

The growth performance is the sum of all the parameters aforementioned. In general, improvements in intestinal morphology and organ development can lead to increase nutrient absorption, which will be reflected in enhanced performance [19,30,37]. As is clear, different carbohydrates from dietary fiber can have different modes of action once ingested by the bird. Therefore, in order to make conclusions about the effect of fiber, there are different factors that need to be closely considered. Factors such as fiber source (i.e., soluble vs. insoluble), particle size, level of inclusion, specie, age, physiological status (i.e., laying hen vs. broiler), dietary energy and protein (i.e., amino acids) levels, and duration of inclusion are among the most influential factors determining the effects of fibers on broiler diets [3,12,13,26,28,69]. Most studies report changes in performance when insoluble fiber is included in the diets (Table 4).

In general, inclusion of insoluble fiber such as oat hulls, wood shavings, and soyhulls has been shown to increase the feed efficiency between 3–5% and increase body weight between 2–5% when included at 3–5% in the diet [15,21,28]. It is important to point out that many of the papers herein (Table 4) cited did not formulate isonitrogenous and isocaloric diets. This might be one of the reasons behind the differences observed in the results obtained when using dietary fiber. On the other hand, inclusion of soluble fiber such as high-methylated pectins reduced feed efficiency up to 28% when provided in diets at 3% [10]; soluble fiber such as guar gum decreases feed efficiency by 4% when fed at 0.3% in the diets [42]. Another reason is the difference in fiber type composition as shown in Table 5.

The insoluble portion of the plant cell wall is tri-dimensionally arranged in fibrillar polysaccharides such as cellulose, hemicellulose, and/or encrusting non-saccharide substances such as lignin [2]. Predominantly, the insoluble fiber of the cell walls is associated with other polysaccharide matrices of pectic carbohydrates, conferring different structural and functional characteristics depending on their amounts [82]. Because of this intrinsic chemical and structural organization, it is hard to separate soluble from insoluble NSP in feedstuffs, and it is important to understand both fractions individually and in conjunction when formulating diets for poultry species. The ratios of insoluble and soluble components can vary based on grain type, cultivar, environmental conditions, and other associated factors (Table 5). In general, water insoluble NSP contain long sequences of β-1,4 glycosidic units. The solubility of a polysaccharide is determined by the intramolecular (i.e., saccharide-saccharide interaction within molecule) and molecule-water interactions. For insoluble polysaccharides, the intramolecular interactions are higher, including more hydrogen bonding. Insoluble fiber components include cellulose, hemicellulose, and lignin [7,83]. Soluble fiber is found in association with insoluble fiber mainly as xyloglucan-cellulose and xyloglucan-pectic polysaccharides [84] (Table 5). The tri-dimensional structure of soluble fiber is referred as matrix polysaccharides, which includes mainly arabinoxylans, β-glucans, and pectin [2]. The soluble carbohydrates, including oligosaccharides and polysaccharides, are the most influential in terms of growth performance, nutrient absorption modulation, and intestinal welfare. In general, water-soluble or partially water soluble NSP have β-1,4 glycosidic linkage backbones with β-1,3 linkages. The degree of solubility is associated with the degree of branching of the NSP molecule.

### 2.6. Intestinal Health

**Figure 1:**
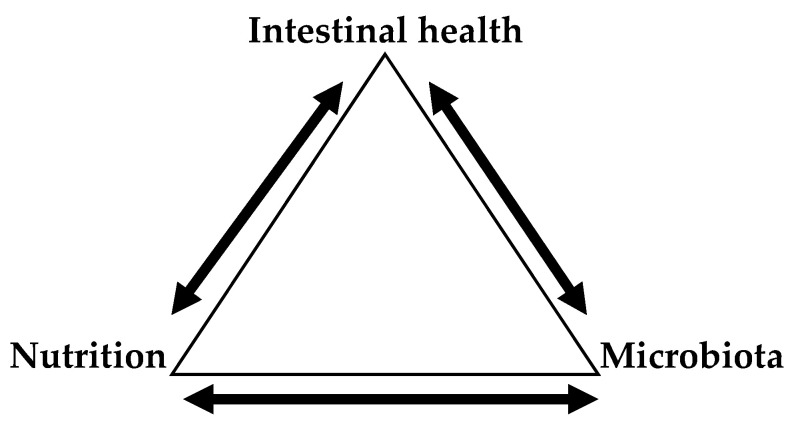
Interrelationship between intestinal health with nutrition and microbiota.

Since the initial removal of antibiotics from poultry diets, intestinal health has been one of the most critical topics that poultry nutritionists have dealt with [85]. This is because the gut contains more than 600 species of bacteria and more than 20 hormones associated with endocrine, paracrine and autocrine modulation; it also digests and absorbs nutrients [86] and consumes about 20% of the incoming energy [87]. Besides those important roles, the gut accounts for a substantial amount of the body’s immune cells, being critical in terms of overall animal health [88,89]. Thus, feed efficiency and overall animal health are dependent of intestinal health. Because of its ability to scape digestion and absorption, DF can affect intestinal health directly by functioning as a direct source of energy and extra nutrients or indirectly by causing the modulation of intestinal microbiota and, subsequently, gut functionality [53]. The extent to which dietary fiber can positively or negative affect intestinal health is based on solubility and fermentability [46,47]. In the case of insoluble fibers that are composed of insoluble β 1–4/1–6 chains (i.e., cellulose, hemicellulose, lignin) (Table 5), they cannot be utilized by the intestinal microbes to a substantial extend and, therefore, their total effect in intestinal health has been regarded to be limited [2]. However, it has been pointed out the possibility of intestinal upregulation associated to a healthy gut with the presence of insoluble fiber fractions [86]. Soluble dietary fiber, on the other hand, has a more profound effect in terms of intestinal health. The major components of soluble fibers found in commonly used feedstuffs are β-glucans, arabinoxylans, and pectins [2]. The presence of barley and rye β-glucans (β 1–3, 1–4), and wheat arabinoxylans has been clearly proven to increase intestinal problems and presence of necrotic enteritis in poultry species [90,91,92]. Viscous soluble fibers act directly by reducing intestinal passage rate which allows the colonization and establishment of undesirably bacteria in the intestines [39]. They can also interact with the surface of the intestinal epithelial to increase mucin secretions that are rich in nutrients that facilitate and promote bacterial growth [93]. It is clear that the results of feeding diets containing high levels of NSP is closely associated to the initial intestinal microbial status [94]. For instance, when comparing germ-free chickens to their normal counterparts, germ-free seem to lack the effect of viscosity in their performance [10]. However, germ-free animals seem to be more susceptible to intestinal infections due to the lack of competitive exclusion provided by indigenous microbiota [95]. Therefore, intestinal health is directly affected by nutrition and intestinal microbiota (Figure 1).

As shown in Figure 1, there is a close bidirectional relationship between intestinal health-nutrition, and intestinal health-microbiota. Intestinal health affects the efficiency with which the nutrients are absorbed having direct effects that may affect several systemic functions [96]. On the other side, the quality of feedstuffs and/or nutrients being provided determine digestibility and subsequent partitioning of digested nutrients among different tissues (including intestines) and the overall health of the animal. For instance, feeds high in viscous fibers increase the mucus secretions reducing the nutrient digestibility (metabolizable energy, protein) that is what we call “poor digestibility” [97]. The interrelationship between intestinal health and microbiota is noted by the fact that fully stablished microbiota in older animals makes them more resistant to disturbances compared to younger animals [86]. Awad et al., [98], reported that birds younger than two weeks had more *Proteobacteria* (increase pro-inflammatory cytokines), whereas *Firmicutes* and *Tenericutes* (increase anti-inflammatory cytokines) dominated in birds older than two weeks. Nutrition and microbiota share a very tight interrelationship with each other. With the removal of antibiotics, we have realized that we are not only feeding the animal, but the gut as well. In this way indigested fibers and other nutrients can be used by harmful bacteria that causes dysbiosis or by commensal bacteria that can yield short-chain fatty acids (SCFA) that are utilized by the animal [99] and some of them (i.e., butyrate) are associated to intestinal health [100]. Therefore, at the end, the type of fiber (solubility and fermentability) determines the type of bacteria that dominates the gut and the immune response the host will activate in response to such changes.

## 3. Current Unknowns

1. A typical broiler diet is composed of about 70% carbohydrates which includes starch, oligosaccharides, and NSP. The oligosaccharide and NSP is the least known and understood groups but their accurate determination is required in order to be able to determine their physiological functions. Crude fiber is the most commonly used method for the determination of fibrous components in broiler and poultry diets. This method separates fibrous components using weak acids and bases and the results obtained include portions cellulose and lignin ignoring oligosaccharides, pectins and hemicellulose which play a crucial role in intestinal functioning, nutrient digestion and intestinal microflora modulation. Therefore, a more accurate determination of total dietary fiber is paramount to be able to accurately measure physiological responses.
2. Dietary fiber, more often than not, influence intestinal morphology, increasing villi height and the overall size of the intestinal tract. Energy expenditure by the gastrointestinal tract can account up to 20–30% of that of the entire body. Therefore, it is crucial to be able to determine how those changes in intestinal morphology affect the overall requirements not for energy only but for amino acids associated with intestinal growth.
3. Further intestinal epithelial turnover has been observed in species other than poultry which leads to the question, what is the extend of protein turnover (abrasion) caused by dietary fiber and how to offset this problem? There should be a threshold of dietary fiber that can exert the positive effects of nutrient absorption and intestinal development without charging the bill of increased endogenous loses. In order to determine this, it is important to be able to accurately determine the effect of each fiber component from a chemical and physiological view.

## 4. Conclusions and Future Directions

Dietary fiber is an intrinsic component in cereal grains and oilseeds used in the formulation of broiler and poultry diets. Both insoluble and soluble fiber components have direct effects in intestinal morphology, organ growth, nutrient utilization, and microflora modulation, to different extents. The results obtained when using dietary fiber relies on factors such as fiber type, inclusion level, particle size, and diet formulation. The insoluble fibers are regarded as functional nutrients because of their ability to scape digestion and modulate nutrient digestion and general intestinal parameters. Because of their insolubility, they have minimal or no effect on the intestinal microflora with significant effects in intestinal development and nutrient digestibility when used in amounts between 3–5% in the diet. On the other hand, the group of soluble fibers has been regarded as antinutrients because of their hygroscopic properties and their ability to modulate intestinal functionality whether directly or indirectly through microbial changes. The presence of soluble fibers such as pectin and arabinoxylans can substantially impact the accessibility of the intestinal enzymes to substrates, resulting in lower nutrient release and subsequent nutrient digestibility. Nevertheless, it is important to emphasize that not all water-soluble fibers are antinutritional and that low molecular weight water-soluble carbohydrates such as mannan-oligosaccharides, inulin, and other prebiotics play an important role in the modulation of intestinal microflora and potential immune response. Insoluble as well as soluble carbohydrates are found in cereal grains and oilseeds, and this is thus something that poultry nutritionist have to deal with during diet formulation. To accurately determine the positive as well as the “unseen” negative effects of dietary fiber, it is important to have an accurate determination of fiber from feed ingredients using more adequate methodologies that may allow for the assessment of saccharides. Finally, to be able to make correct use of fibrous feed ingredients is paramount to carry out experiments that would assess the metabolic impact of dietary fiber on the additional requirements of energy and other nutrients (i.e., amino acids) used in order to be able to compensate for organ and tissue growth and to, finally, introduce such corrections into the nutrient matrix.

## Figures and Tables

**Table 1:** Physiological response of different poultry species to dietary fiber.

Specie	Ingredient ^1^	Effects ^2^	% ^3^	Age/Duration ^4^	Reference
Broilers	Oat hulls, sugar beet pulp	↑ RW of proventriculus and ceca; oat hulls ↑ RW of the gizzard.	3%	1 day-old/21 days	[3]
Broilers	Sugar beet pulp and rice hulls	Sugar beet pulp ↑ RW of jejunum and ileum, whereas rice hulls ↓ RW; rice hulls ↑ jejunal villi height.	3%	1 day-old/42 days	[6]
Turkeys	Mix of sunflower meal and soyhulls	↑ villi height and morphology parameters inconsistently in duodenum, jejunum, and ileum.	3, 6, and 9% CF	1 day-old/98 days	[13]
Quail	Wheat fiber	↓ RW of the liver, ↑ RW and villi:crypt ratio of duodenum, jejunum, and ileum at 1.5%.	0, 0.5, 1, and 1.5%	1 day-old/28 days	[14]
Geese	Alfalfa, barley hulls, rice hulls, cellulose, lignin, or pectin	↑ in villi height with alfalfa meal, rice hulls or pectin and reductions with lignin.	Vary	14 days-old/28 days	[25]
Broiler	Soyhulls and cellulose	Soyhulls ↑ duodenal, jejunal, and ileal villus height.	2–8% CF	1 day-old/20 days	[26]
Broiler	Wood shavings	↑ RW of proventriculus and gizzard; ↓ RW of small intestine.	6%	1 day-old/21 days	[28]
Broilers	Oat hulls, soyhulls	↑ RW of proventriculus and gizzard; ↓ RW of small intestine.	3%	1 day-old/21 days	[21]
Broilers	Inulin	↑ villi height either at 0.5 or 1%.	0.5, 1%	1 day-old/42 days	[29]
Broilers	Oat and barley hulls	↑ RW of gizzard and of intestines.	15%	1 day-old/17 to 32 d-of-age	[30]
Broilers	Pectin and beet pulp	Pectin ↓ the liver weight.	1.5 and 3%	1 day-old/6–27 days	[31]

^1^ Indicates the ingredient that was used as the main source of dietary fiber. ^2^ ↑ = increased; ↓ = decreased; RW = relative weight. ^3^ Indicates the net % of the ingredient added to the diet; when percent is followed by crude fiber (CF) indicates that the ingredient was added to achieve that level of crude fiber. ^4^ Indicates the age of the poultry specie when the experiment was started. Duration indicates the duration of the experiment.

**Table 2:** Impact of fiber type and amount on nutrient digestibility.

Specie	Ingredient ^1^	Effects ^2^	% ^3^	Iso-Nutrient ^4^	Age/Duration ^5^	Reference
Broilers	Oat hulls	↑ the TAR of dry matter, organic matter, nitrogen, ether extract, and amen.	3%	No	1 day-old/21 days	[3]
Broilers	Oat hulls	↑ starch digestibility.	10%	No	11 day-old/22 days	[15]
Broilers	Soyhulls and cellulose	Soyhulls ↑ amino acids digestibility.	2–8% CF	Yes	1 day-old/20 days	[26]
Broilers	Cellulose	9% ↑ starch digestibility.	6%	No	1 day-old/21 days	[28]
Broilers	Oat hulls	↑ TTAD of dry matter, nitrogen and ether extract digestibility.	3%	Yes	1 day-old/21 days	[21]
Broilers	Oat and barley hulls at 50:50, wt:wt; coarse and fine	↓ AMEn digestibility, and ↑ starch digestibility.	15%	No	1 day-old/18 to 32 d-of-age	[30]
Broilers	Oat hulls	10% oat hulls ↓ AMEn but ↑ starch digestibility.	4, 10%	No	7 day-old/14 days	[41]
Broilers	Guar gum	↓ AD of lipids, starch, protein, and AMEn at 1 and 3 g/kg.	1 or 3 g/kg diet	Yes	7 day-old/14 days	[42]
Broilers	Pectin from citrus pulp	↑ AME and AMEn with levels of pectin; quadratic ↓ in dry matter digestibility; ↓ in nutrient digestibility.	1, 3, 5%	Yes	1 day-old/31 days	[43]
Broilers	Cellulose	↑ Arginine, and Valine digestibility.	3, 8% CF	No	1 day-old/21 days	[27]

^1^ Indicates the ingredient that was used as the main source of dietary fiber. ^2^ ↑ = increase/improvement; ↓ = decrease/impairment; TAR = total apparent retention; TTAD = total tract apparent digestibility; AD = apparent digestibility AMEn = nitrogen-corrected apparent metabolizable energy. ^3^ Indicates the net % of the ingredient added to the diet; when percent is followed by crude fiber (CF) indicates that the ingredient was added to achieve that level of crude fiber. ^4^ Yes: indicates diets formulated to be isonitrogenous and isocaloric. No: indicates diets formulated with variable nutrient content. ^5^ Indicates the age of the poultry specie when the experiment was started. Duration indicates the duration of the experiment.

**Table 3:** Influence of dietary fiber on intestinal microflora in poultry species.

Specie	Ingredient ^1^	Effects ^2^	% ^3^	Age/Duration ^4^	Reference
Quail	Wheat fiber	No effects.	0, 0.5, 1, and 1.5%	1 day-old/28 days	[14]
Broilers	Inulin	↑ bifidobacteria and decrease E. Coli counts in cecal contents.	0.5, 1%	1 day-old/42 days	[29]
Broiler	Mix DDG and wheat	↑ Selenomonadales, Enterobacteriales, and Campylobacterales.	6 (starter) and 8% (grower)	1 day-old/21 days	[52]
Laying hen	Mix DDG and wheat	No changes.	6 (starter) and 8% (grower)	1 day-old/21 days	[52]
Broilers	Rye or pectin	Ileal segments had 2 or 3-log higher counts compared to control group.	4.50%	1 day-old/14 days	[58]

^1^ Indicates the ingredient that was used as the main source of dietary fiber. DDG = dried distillers’ grains. ^2^ ↑ = increase/improvement; ↓ = decrease/impairment. ^3^ Indicates the net % of the ingredient added to the diet; when percent is followed by crude fiber (CF) indicates that the ingredient was added to achieve that level of crude fiber. ^4^ Indicates the age of the poultry specie when the experiment was started. Duration indicates the duration of the experiment.

**Table 4:** Influence of dietary fiber type on growth performance.

Specie	Ingredient ^1^	Effects ^2^	% ^3^	Iso-Nutrient ^4^	Age/Duration ^5^		Reference
Broilers	Oat hulls, sugar beet pulp	Oat hulls ↑ daily ABW by 7.6%.	3%	No	1 day-old/21 days		[3]
Broilers	Sugar beet pulp	↓ FE by 9%.	3%	No	1 day-old/42 days		[6]
Broilers	Oat hulls	↑ FE by 3%.	10%	No	11 day-old/22 days		[15]
Broilers	Oat hulls	10% oat hulls ↓ FE by 6%.	4 and 10%	No	7 day-old/14 days		[12]
Turkey	Sunflower meal and soyhulls	6% CF ↑ 2.5% BW; 9% CF ↓ FE by 3.8%.	3, 6, 9% CF	Yes	1 day-old/98 days		[13]
Quail	Wheat fiber	↑ BW by 5% and ↑ FE by 5% at 1.5% in the diet.	0, 0.5, 1, and 1.5%	No	1 day-old/28 days		[14]
Broilers	Soyhulls and cellulose	↑ FE by 8% compared to cellulose.	2–8% CF	Yes	1 day-old/20 days		[26]
Broilers	Wood shavings	↑ FE by 4.7%.	6%	No	1 day-old/21 days		[28]
Broilers	Oat hulls, soyhulls	↑ FE by 3.8%.	3%	Yes	1 day-old/21 days		[21]
Broilers	Inulin	↑ BWG by 8% from 25–42 days when at 1% in the diet.	0.5, 1%	Yes	1 day-old/42 days		[29]
Broilers	Oat and barley hulls	Fine hulls ↓ FE by 4.7%; coarse ↑ BWG by 2%.	15%	No	1 day-old/17 to 32 days-of-age		[30]
Broilers	Guar gum	↓ FE by 4% when fed at 3 g/kg.	1 or 3 g/kg diet	Yes	7 day-old/14 days		[42]
Broilers	Pectin and beet pulp	Pectin ↓ BWG by 28% and FE by 28% when fed at 3%.	1.5 and 3%	Yes	1 day-old/6–27 days		[31]

^1^ Indicates the ingredient that was used as the main source of dietary fiber. ^2^ ↑ = increase/improvement; ↓ = decrease/impairment; BW = body weight; ABW = average body weight; FE = feed efficiency; BWG = body weight gain. ^3^ Indicates the net % of the ingredient added to the diet; when percent is followed by crude fiber (CF) indicates that the ingredient was added to achieve that level of crude fiber. ^4^ Yes: indicates diets formulated to be isonitrogenous and isocaloric. No: indicates diets formulated with variable nutrient content. ^5^ Indicates the age of the poultry specie when the experiment was started. Duration indicates the duration of the experiment.

**Table 5:** Fiber type and composition of the ingredients used in the literature cited in Table 1, Table 2, Table 3 and Table 4.

Ingredient	IF ^1^, %	SF ^1^, %	Major NSP ^2^	Structure-Linkages	Reference
Oat hulls	83.3	1.7	Cellulose/lignin	Glu β 1–4/β-O-4	[70]
Beet pulp	1.9–3	28	Uronic acid	β 1–4	[71]
Rice hulls	87.3	2.7	Cellulose/arabinoxylan/lignin	Glu β 1–4/β-O-4	[72]
Sunflower meal	11.3	3.9	Xylose/uric acid	β 1–4	[73]
Wheat fiber	44.9	7.6	Cellulose	Glu β 1–4	[71]
Alfalfa meal	46.7	7.9	Cellulose/lignin	Glu β 1–4/β-O-4	[74]
Barley hulls	20.3	9.8	Cellulose/hemicellulose/lignin	Glu β 1–4/β-O-4	[75]
Cellulose	97	2.3	Cellulose	Glu β 1–4	[76]
Pectin	0	65.4	Uronic acid	β 1–4	[76]
Soyhulls	49.3	13.3	Pectin/galacturonic acid	galacturonic acid 1α→4 linkages	[77]
Wood shavings	91.7	-	Cellulose/lignin	Glu β 1–4/β-O-4	[15]
Inulin ^3^	-	>90	Fructose units	β 2–1	[29,78]
Guar gum	26	32	Mannose/galactose	β 1–4/1–6	[79]
DDGS ^4^	25.5	3.4	Arabinoxylan	β 1–4	[80]
Wheat	9.3	1.9	Arabinoxylan	β 1–4	[80,81]
Rye	11	4.2	Arabinoxylan	β 1–4	[73]

^1^ Indicates that the insoluble fiber (IF) and soluble fiber (SF) content was extrapolated using contents of acid detergent and neutral detergent fibers. ^2^ NSP = non-starch polysaccharides. ^3^ From the original source (seed endosperm or leaves). Authors don’t mention the source of inulin; it is accepted that most inulin is fermented by intestinal microbial (soluble) [78]. ^4^ DDGS = Dried distillers’ grains with solubles.

## Data Availability

Not applicable.
